# Insertion of Short Amino-Functionalized Single-Walled Carbon Nanotubes into Phospholipid Bilayer Occurs by Passive Diffusion

**DOI:** 10.1371/journal.pone.0040703

**Published:** 2012-07-16

**Authors:** Sebastian Kraszewski, Alberto Bianco, Mounir Tarek, Christophe Ramseyer

**Affiliations:** 1 Laboratoire de Nanomédecine, Imagerie et Thérapeutique, EA 4662, Université de Franche-Comté, Centre Hospitalier Universitaire de Besançon, Besançon, France; 2 CNRS, Institut de Biologie Moléculaire et Cellulaire, Laboratoire d’Immunologie et Chimie Thérapeutiques, Strasbourg, France; 3 Structure et Réactivité des Systèmes Moléculaires Complexes, Equipe de Chimie et Biochimie Théoriques, UMR 7565, CNRS, Université de Lorraine, Nancy, France; University of Akron, United States of America

## Abstract

Carbon nanotubes have been proposed to be efficient nanovectors able to deliver genetic or therapeutic cargo into living cells. However, a direct evidence of the molecular mechanism of their translocation across cell membranes is still needed. Here, we report on an extensive computational study of short (5 nm length) pristine and functionalized single-walled carbon nanotubes uptake by phospholipid bilayer models using all-atom molecular dynamics simulations. Our data support the hypothesis of a direct translocation of the nanotubes through the phospholipid membrane. We find that insertion of neat nanotubes within the bilayer is a “nanoneedle” like process, which can often be divided in three consecutive steps: landing and floating, penetration of the lipid headgroup area and finally sliding into the membrane core. The presence of functional groups at moderate concentrations does not modify the overall scheme of diffusion mechanism, provided that their deprotonated state favors translocation through the lipid bilayer.

## Introduction

Highly ordered carbon-based nanomaterials such as fullerenes and carbon nanotubes (CNTs) possess unique structural, mechanical, and electronic properties suited for numerous applications. [1], [2], [3], [4] In the biomedical field, due to their high hydrophobic and lipophilic characters, carbon nanomaterials have the capacity to easily penetrate cell membranes. Thus, they are of great interest for the intracellular delivery of therapeutic proteins, peptides, genes and drugs. [5],[6],[7],[8],[9],[10],[11] The mechanisms of CNT uptake into mammalian cells is still not fully elucidated, and sometimes conflicting results are presented. Even if it was already shown that this disagreement is apparently due to the difference of tested samples, [12] the cellular uptake of carbon nanotubes functionalized with small moieties looks independent of functional groups and cell types and could be rather attributed to the differences in their physical properties like nanotube length and diameter. [13] It seems that the short and amino functionalized nanotubes (*i.e.* hundreds of nm in length) could act as tiny and straight “nanoneedles” able to passively penetrate the cell membrane. [9] Alternatively, carbon nanotubes modified with proteins or DNA sequences display an energy-dependent endocytotic route of penetration. [7], [14] However, Pantarotto *et al.* observed passive diffusion of peptide functionalized single-walled carbon nanotubes (*f*-SWNTs) through the cell membrane. [15] Furthermore, *f*-SWNTs showed similar behavior when their incubation with cells is carried out at low temperature, [13] or when treated with sodium azide, a well-known endocytosis inhibitor. [12] By contrast, endocytosis was clearly identified when acid oxidized or coated via non covalent adsorption SWNTs (hundreds of nm in length and 1 to 5 nm in diameter) were used as intracellular transporters for proteins and DNA. [7], [14], [16] Phagocytosis was also proposed as an uptake mechanism in mouse peritoneal macrophages incubated under various concentrations of SWNTs (∼1 nm diameter, and ∼1 µm length) dispersed by Pluronic surfactant. [17] Finally, multi-walled carbon nanotubes (MWNTs) seems to enter human macrophages actively and passively via incomplete phagocytosis or impaling the membrane. [18] This has been also futher explored and confirmed by the recent study of Lacerda *et al.* [19].

In light of these results, a clear understanding of the molecular interaction between *f*-SWNTs and the cell membrane is necessary. So far, only a few theoretical studies have been carried out using dissipative particle dynamics, coarse-grained or short constrained all-atom molecular dynamics (MD) simulations of single non functionalized SWNTs with model membranes. [20], [21], [22], [23], [24], [25], [26] The main results are that: i) the entry of closed SWNTs is favored when the tube is flat on the membrane surface, ii) the global minimum is reached when SWNT is fully embedded in the lipid bilayer, and iii) increasing SWNT length reinforce the preference for horizontal embedding in the core of the cellular membrane. This contrasts with previous coarse-grained simulations where the favored insertion’s orientation of the open ended SWNTs was a perpendicular one with respect to the membrane plane. [20] Finally, it was also shown that opened SWNTs disrupt the membrane structure irreversibly by dragging lipid molecules along with it. [27], [28].

In the present study, we explore the uptake mechanism of different types of SWNTs by a lipid bilayer using unconstrained atomistic MD simulations, in order to determine: i) if the uptake pathway of short SWNTs is a passive diffusion or an endocytosis process, and ii) how functionalization influences the penetration mechanism.

## Results and Discussion

### Uptake of Closed and Functionalized SWNTs

**Figure 1:**
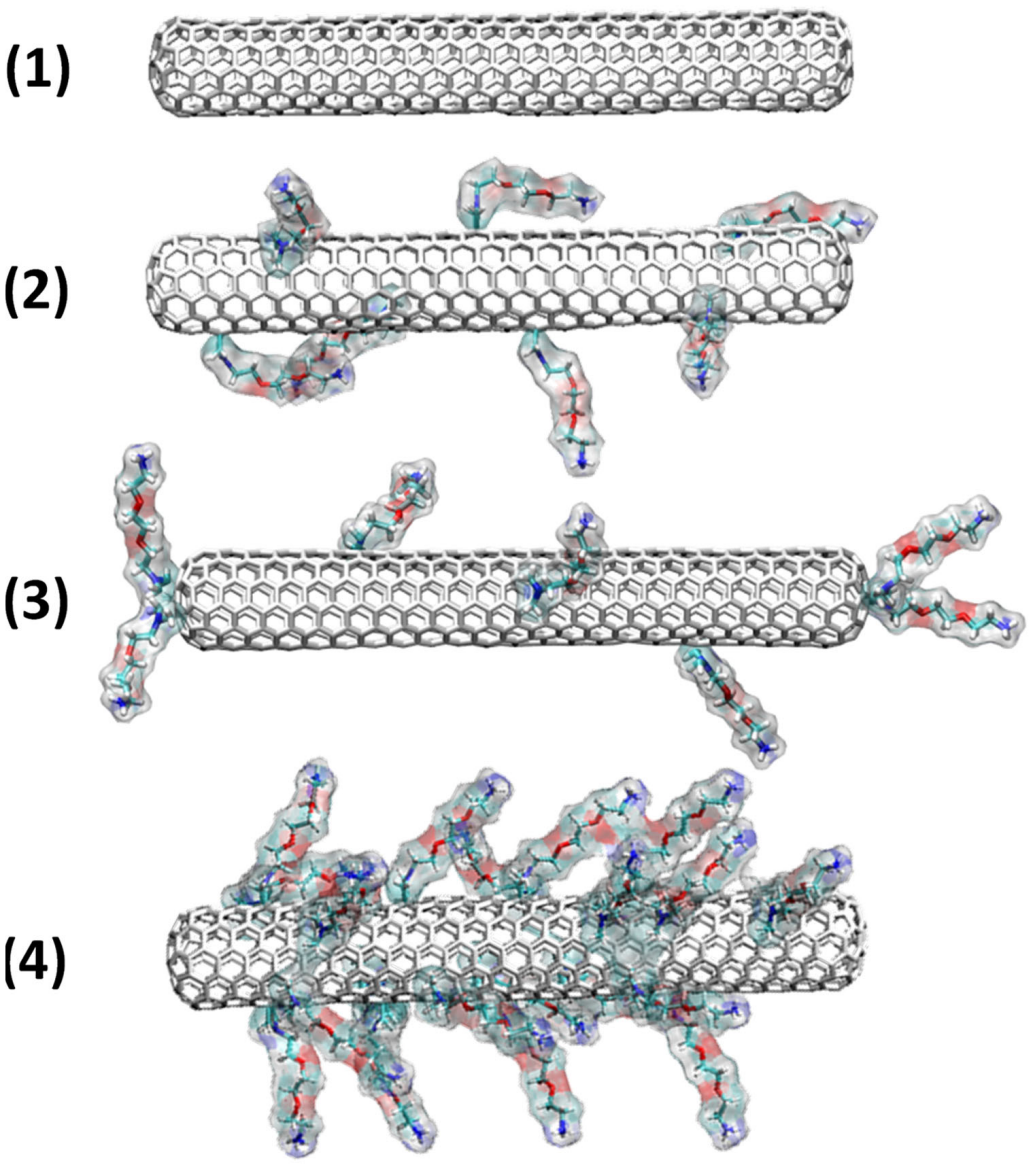
Studied models of closed *f*-CNTs. Different types of closed SWNTs have been investigated depending on their degree of functionalization. Amino derivatives were randomly distributed on the surface of the tubes.

We have performed large scale all-atom MD simulations on various types of non-functionalized and functionalized SWNTs, the largest being on the microsecond time scale, in order to reveal the precise manner by which SWNTs cross the membrane. For this purpose, we have designed a series of SWNT models (Figure 1). SWNTs closed at their tips were tested in four distinct sets: **(1)** non functionalized SWNT, **(2)** low degree surface functionalized SWNT, **(3)** low degree surface and tip functionalized SWNT and **(4)** highly surface functionalized SWNT. Low degree functionalized SWNT has one functional group every 90 carbon atoms, while the highly functionalized one has one functional group every 30 carbon atoms. Here, all functionalized SWNTs bear the water-soluble ammonium groups at the end of a triethylene glycol (TEG) chain (SWNT-[TEG-NH_3_^+^]_n_, – hereafter termed *f*-CNT), randomly dispersed on their surface, as in the case of *f*-CNTs synthesized by the 1,3-dipolar cycloaddition reaction of azomethine ylides. [29] Model **(3)** was considered as the closest to the experimental samples described by Georgakilas *et al*. in which the 1,3-dipolar cycloaddition reaction occurs both at the tips and the sidewall of the nanotubes. [29] The pyrrolidine rings generated during this cycloaddition are located in (1,2) position of the CNT carbon hexagons as well-know from the chemistry on fullerene. [30].

All MD simulations started from configurations where the nanotubes were placed at least 0.5 nm away from the water/membrane interface. Different orientations of the latter (initial angles of 0°, 45° and 90°) with respect to the membrane were considered, in order to check that the penetration mechanism does not depend on the chosen initial conditions. [23].

**Figure 2:**
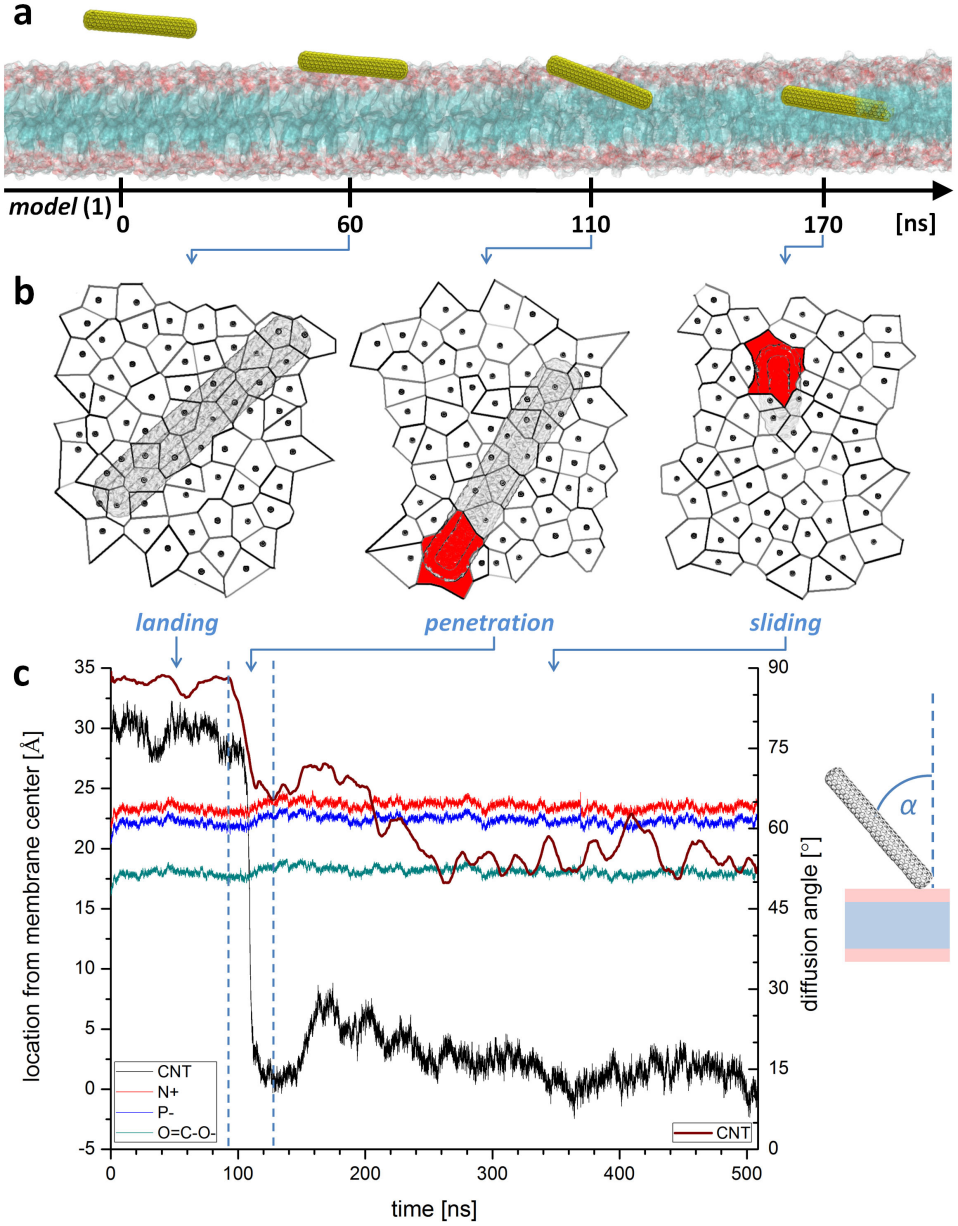
Uptake path of model (1) SWNT. **a**, Internalization mechanism obtained from unconstrained MD simulations with closed and non functionalized SWNT displays a 3-step passive diffusion phenomenon. The lipid membrane head and tails sections are shown as red and blue surfaces, respectively. For clarity, water molecules and counterions are not shown. **b**, Voronoi tessellations of membrane surface present in average an inflation of the area per lipid but reveals also local contractions in the neighborhood of the tube penetrating the membrane. Red areas in Voronoi diagrams correspond to internalizing SWNT. **c**, Close examination of SWNT trajectory (black curve) and insertion angle (wine curve) show sudden penetration phase. Left ordinate scale refer to SWNT center of mass position (black curve), mean nitrogen position of lipid headgroups (red curve), mean phosphorous position of lipid headgroups (blue curve) and mean position of lipid glycerol backbone (green curve). Right ordinate scale refers to SWNT insertion angle (α) with respect to the normal of the membrane plane (wine curve). The angle curve is smoothed by averaging the angle value in 1 ns window.

Figure 2a illustrates the diffusion pathway of a closed and non functionalized SWNT model **(1)** across a POPC phospholipid bilayer (see also Video S1). For all initial configurations, the nanotube penetrates the bilayer by a passive diffusion mechanism without any significant deformation of the membrane that indicates endocytosis. The analysis of the trajectories clearly confirms the “nanoneedle” mechanism, proposed by Kostarelos, Prato and Bianco. [13] At the molecular level, this mechanism can be described by three successive steps. The first concerns the “landing and floating” of the SWNT on the lipid bilayer. This process stops when one or more lipids protrude slightly out of the bilayer to better soak one nanotube extremity, providing therefore for a slight opening of the hydrophilic headgroup region. Then, the SWNT slightly tilts away from the bilayer plane (see wine curve in Figure 2c) and rapidly acts as a “needle” penetrating the membrane through the tip first. [26] This step, which took approximately 10 ns, was then followed by a “sliding” phase where the SWNT simply slides along its axis deeper into the core of the bilayer.

At first glance, no major perturbation of the lipid bilayer arrangement appeared to occur during the nanotube translocation. The thickness of the bilayer remained almost constant during the approach and penetration of the nanotube. A close examination shows however that the bilayer thickness increased by ∼0.2 nm over the whole system (see Figure 2c around 100 ns). Concomitantly, the total area of the system increased during the nanotube penetration. In order to better characterize the perturbation of the bilayer, we have analyzed the lipid-water interface by Voronoi cells associated to the area occupied by each lipid (Figure 2b). The calculation reveals that in the neighborhood of SWNT, area per lipid decreases from its equilibrium value during immersion, as recently observed using coarse grained model. [28].

**Figure 3:**
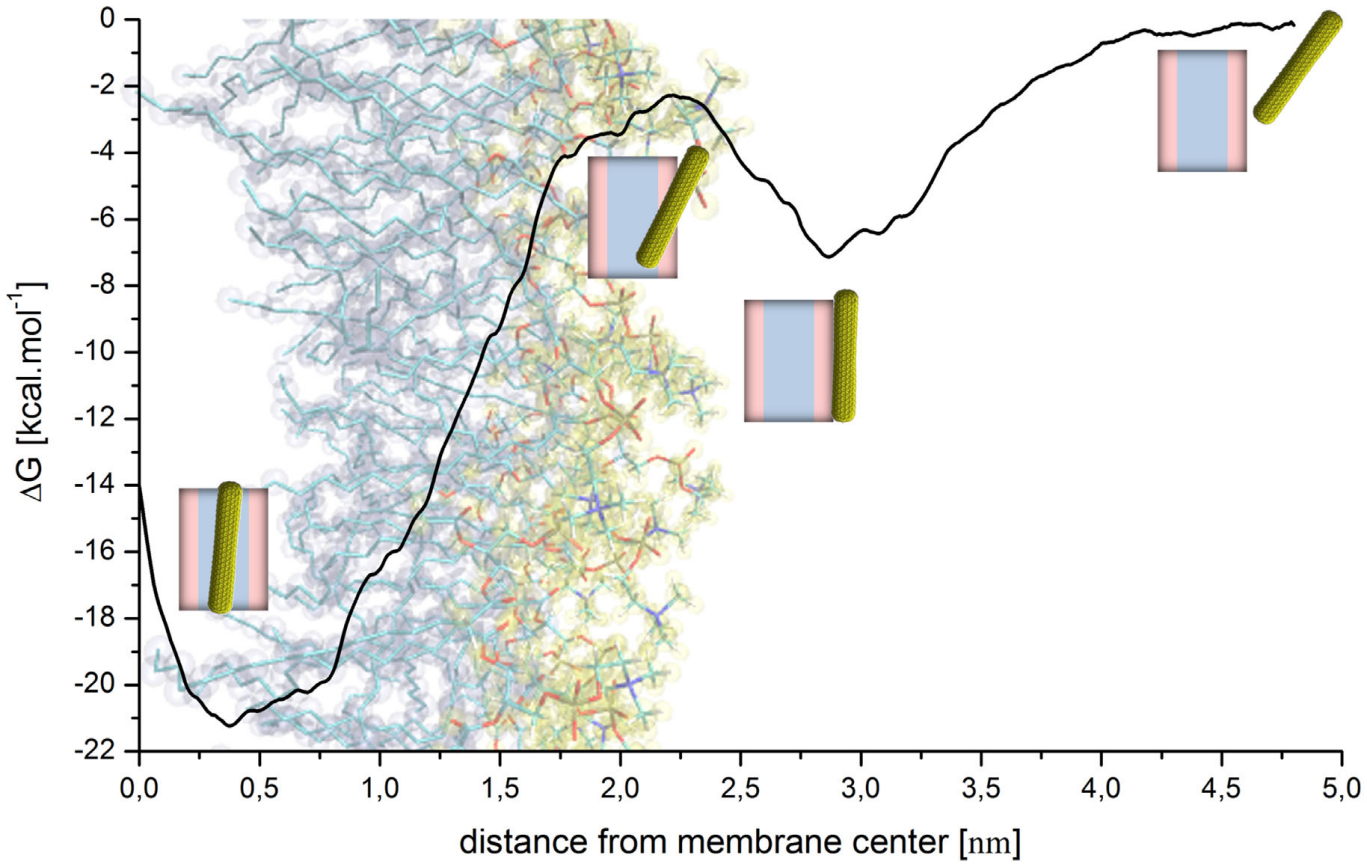
Free energy profile of model (1) SWNT insertion. The profile obtained using the ABF approach of a closed and pristine SWNT diffusing across a POPC bilayer shows two energy minima: One at the lipid/water interface and another, more attractive in the bilayer midplane.

We probed the free energy profile of this non functionalized closed SWNT moving across the bilayer. In principle, this is a multidimensional problem since one needs to sample both the translocation of the nanotube and various orientations with respect to the lipid membrane. [20] For computational reasons, we have chosen here instead to evaluate the free energy profile sampled along approximately the same pathway undertaken during the unbiased MD simulations. This profile was calculated along the normal to the membrane surface as a function of the distance between the center of the SWNT and the center of the lipid bilayer using the adaptive biasing force (ABF) method. [31], [32] All regions corresponding to the landing and floating, penetration and sliding phases were explored. The profile shown in Figure 3 exhibits an attractive potential well of −7.1 kcal/mol located at the water/lipid interface reflecting the attraction of the SWNT to the lipid headgroups during the landing and floating of the SWNT on the lipid surface. The 4.9 kcal/mol barrier occurring at the polar headgroups of the bilayer, represents the energetical cost for the unfunctionalised 5 nm long SWNT to tilt and push away the closest lipid headgroups to allow the SWNT to penetrate the membrane afterwards. One should note that this barrier certainly depends on the tube diameter and length. A rough estimate from the present calculation is ∼1 kcal/mol per nanometer and per ∼1 nm in diameter.

The lipophilicity of the SWNT appears as a deep well of −21 kcal/mol in the energy profile. It corresponds to the attraction of the SWNT to the lipid tails. Such an attractive energy of insertion corresponds with recent theoretical work, especially those of thin and hydrophobic CNTs. [33] Our findings are comparable to recent equilibrium values of CNT translocation up to the bilayer midplane. Finally, the translocation toward the lower leaflet lipid/water interface seems to proceed with a cost of at least 7.5 kcal/mol. This value strongly depends on the tube orientation.

**Figure 4:**
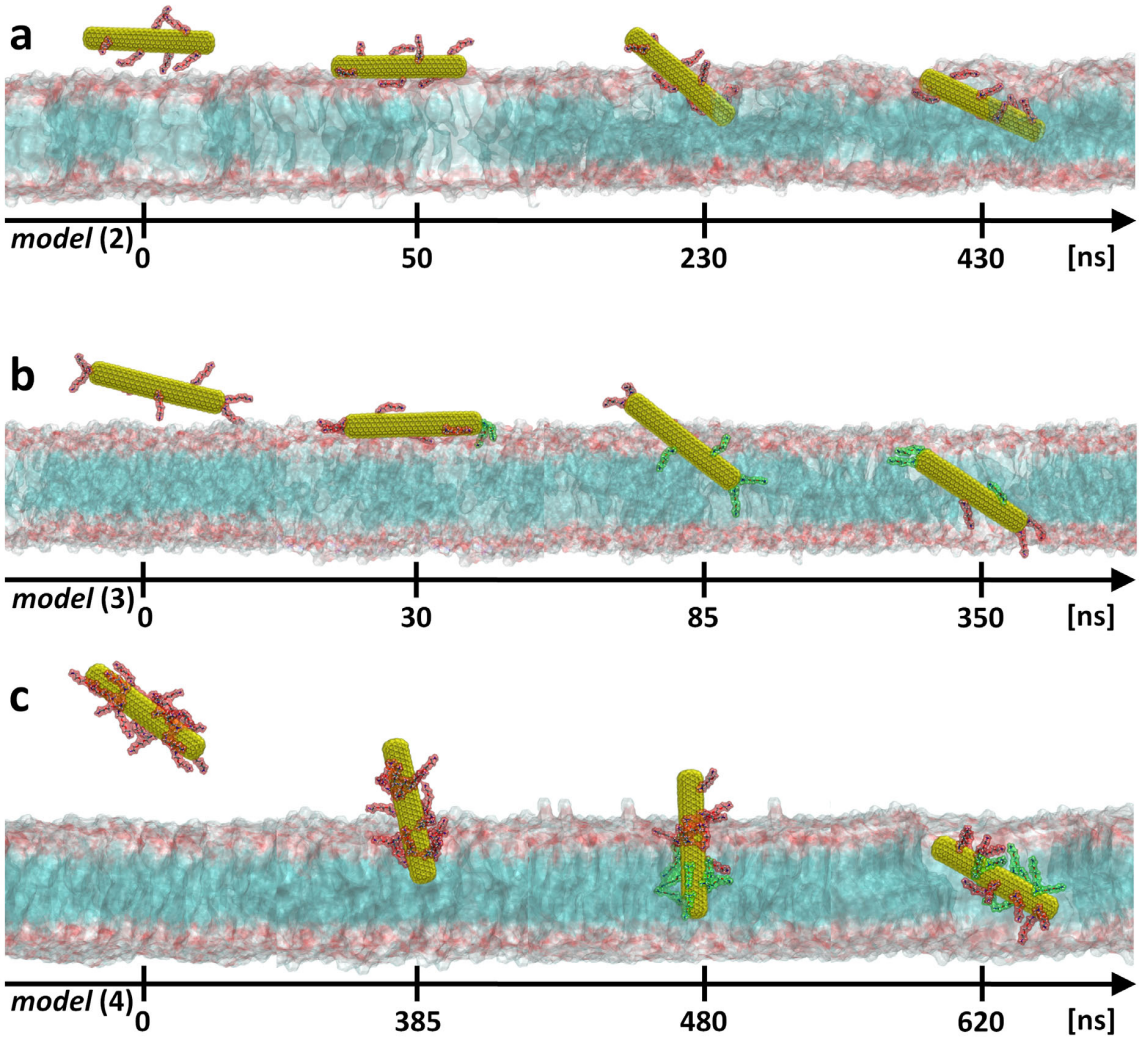
Uptake path of closed *f*-CNTs. Results obtained from unconstrained MD simulations for: **a,** closed and low degree side functionalized SWNT [model **(2)**]; **b,** low degree side and tip functionalized SWNT [model **(3)**]; **c,** or highly side functionalized SWNT [model **(4)**]. Note that *f*-CNTs can be completely taken up only when the cationic functional groups are deprotonated (*cf.* text). The yellow surface represents the SWNT core while the amino functional groups attached to the latter are shown as red (charged form) or green (neutral form) atoms. The lipid membrane head and tails sections are shown as pale red and blue surfaces, respectively. For clarity, water molecules and counterions are not shown.

In order to be as close as possible to the experimental conditions, we have considered a set of water-soluble amino-functionalized SWNTs (Figure 1, models **(2)**, **(3)** and **(4)**). Long MD runs were conducted within the same setups used previously for the neat nanotube. Figure 4 exhibits different snapshots of the uptake process of these *f*-CNTs (see also Videos S2 and S3) and more quantitative views are proposed in Figures S1, S2 and S3. The main feature is that the functional groups do not seem to influence the overall nanoneedle-like process already observed for the neat SWNT. The closed *f*-CNTs are still taken up by the POPC bilayer in a passive way. However, the separate steps of the uptake process were slightly dependant on the degree of functionalization. Precisely, only the lightly functionalized SWNTs seem to undergo the parallel-to-membrane “landing and floating” phase (see Figure S1 and S2). For the highly functionalized one, this phase disappears completely, and is mostly a “perpendicular penetration”. Note that this doesn’t seem to accelerate the overall process (see Figure S3). We found also that the pure “penetration” phase can depend on the degree of functionalization of SWNTs. The higher the density of functional groups attached to the SWNT surface is, the more the penetration angle (with respect to the surface of the membrane) is pronounced (see wine curve in Figures S1c, S2c and S3c). Indeed, MD simulations showed that lightly functionalized SWNTs **(2)** and **(3)** penetrate the membrane at grazing incidence angle while highly functionalized SWNT **(4)** present an almost perpendicular penetration angle. Moreover, in the case of model **(2)** and **(4)** the uncovered *f*-CNT edges appear to be more attracted by the hydrophobic part of the membrane. The results show also that both models rotate towards vertical alignment during penetration phase as it was already observed before. [23], [26] Surprisingly, model **(3)**, which matches better the experimental samples, is taken up more efficiently than the other *f*-CNTs. The neat edges, which promote penetration in case of model **(2)** and **(4)**, are now covered by functional groups and we may expect that they will prevent *f*-CNT from insertion. However, this is not the case. These amino groups immerse deeply between lipid headgroups once the *f*-CNT is landed on the membrane surface (see Figure 4b at 30 ns) moving away the lipid headgroups close to the tip. Such a behavior strongly induces a local membrane surface perturbation, which could be at the origins of the penetration phase. The comparison between the Voronoi cells of models **(1)** and **(3)** shows that just underneath the SWNT, the space to enter into the lipid headgroups is larger for SWNT model **(3)** than for SWNT model **(1)** (see red areas in Figure 2b and S2b) and consequently the Voronoi cells area reduces accordingly in first shell around this zone.

### Translocation Across the Lipid Tails: Importance of the Deprotonation

At this point, it should be mentioned that after entering the membrane, positively charged ammonium (NH_3_^+^) groups strongly bind to the lipid heads due to electrostatic interactions with the negatively charged phosphate groups and preclude further translocation (within the 100 ns time scale) of the nanotube. In order to determine where this occurs, we have analyzed the interaction between the NH_3_^+^ group and the lipid headgroups. During the landing phase, the NH_3_^+^ groups come at a distance of 4.0±0.3 Å (and sometimes even up to 3.4 Å) from the lipid Phosphorous head (see Figure S4). This is clearly visible for model **(2)** (see Figure 4a at 430 ns) and for model **(4)** (see Figure 4c at 385 ns).

The lipid headgroups are known to present a highly prohibitive barrier to amino groups for crossing the lipid bilayers, as reported for instance for lysine derivatives. [34] On the other hand, recent studies based on local pK_a_ measurements or calculations of the partitioning of amino acid side chains into lipid bilayers reported that it is reasonable to expect that cationic residues can change their protonation state once inserted into the membrane. [34], [35] Accordingly we have here, in agreement with our previous investigation, [36] considered that there is a high probability that the amino derivatives change their protonation state when within the highly charged lipid headgroup area. Practically, we proceeded with the deprotonation scheme on models **(3)** and **(4)** as follow: we simply transformed the NH_3_^+^ group into NH_2_ when NH_3_^+^/nearest phosphorous distance was found less than 3.8 Å for at least 100 ps. This value is chosen in agreement with our quantum calculations (see Methods section). Indeed, we found 3.88 Å with the HF/6-31+G(d,p) model (Hartree-Fock theory using a medium-sized basis set) and 3.78 Å with the b3lyp/6-31+G(d,p) model (DFT approach). Accordingly, we compensated its charges according to the Mulliken partial charge calculation scheme, which resulted in neutral functions (see Methods for more details). The corresponding counterion was also deleted to ensure electro-neutrality and total energy conservation. We then restarted MD simulations from the same configuration. As expected, the deprotonation of the charged NH_3_^+^ groups allowed a deeper penetration of the *f*-CNT toward the core of the membrane. We repeated this procedure for each functional group. As a result, within a total simulation time of 395 ns, the *f*-CNT model **(3)** immersed completely into the membrane. Note that, once the neutral functional group reached the lower leaflet lipid/water interface (NH_3_^+^/nearest phosphorous distance shorter than 3.8 Å for at least 100 ps), we proceeded by re-protonating them. This favored further *f*-CNT translocation across the lipid bilayer. For model **(4)**, we proceeded in a similar manner. This resulted in a translocation of the nanotube in a little less than 780 ns as shown in Figure 4c and S3 (see also Video S3).

### Uptake of Opened Functionalized SWNTs

**Figure 5:**
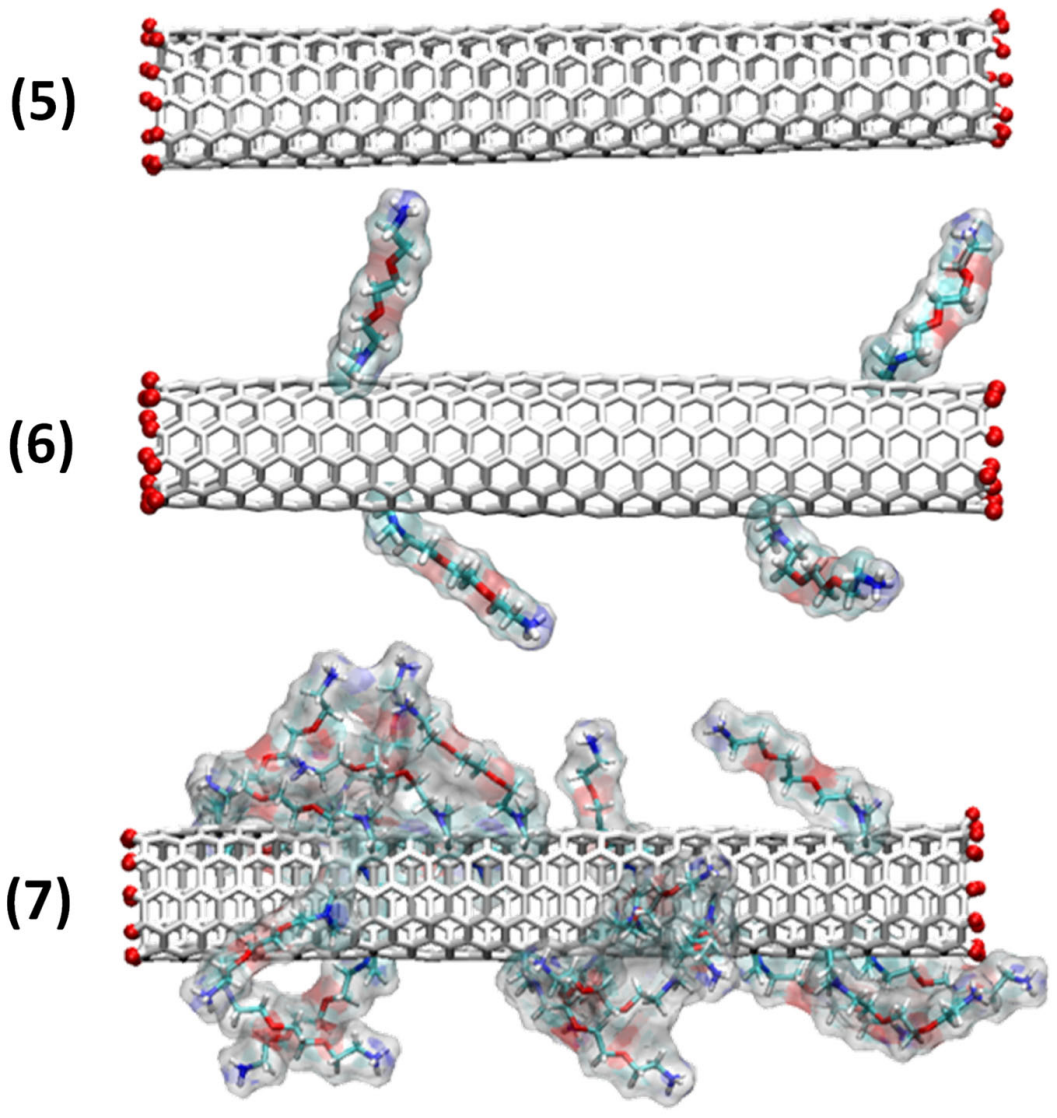
Studied models of open ended *f*-CNTs. Different types of opened SWNTs have been investigated depending on their degree of functionalization. The SWNT edges have been passivated by H atoms. Amino derivatives were randomly distributed on the surface of the tubes.

We have previously reported that it is possible to functionalize also oxidized and shortened carbon nanotubes which are opened at their extremities using the 1,3-dipolar cycloaddition reaction. [13], [37] To be consistent with our study on closed nanotubes, we have thus tested three additional models of opened SWNTs: **(5)** non functionalized, **(6)** low degree functionalized (one functional group every 130 carbon atoms) and **(7)** highly functionalized (one functional group every 30 carbon atoms) (see Figure 5).

The water-soluble ammonium groups were randomly dispersed on the whole surface of the tubes. [29] In addition, the tubes were passivated at their edges with H atoms, which, for simplicity reason, were chosen over the carboxylic functions present at the tips when the tubes are opened. Even if this approach is different from the experimental situation we would only focus on the comparison between opened and closed tubes.

**Figure 6:**
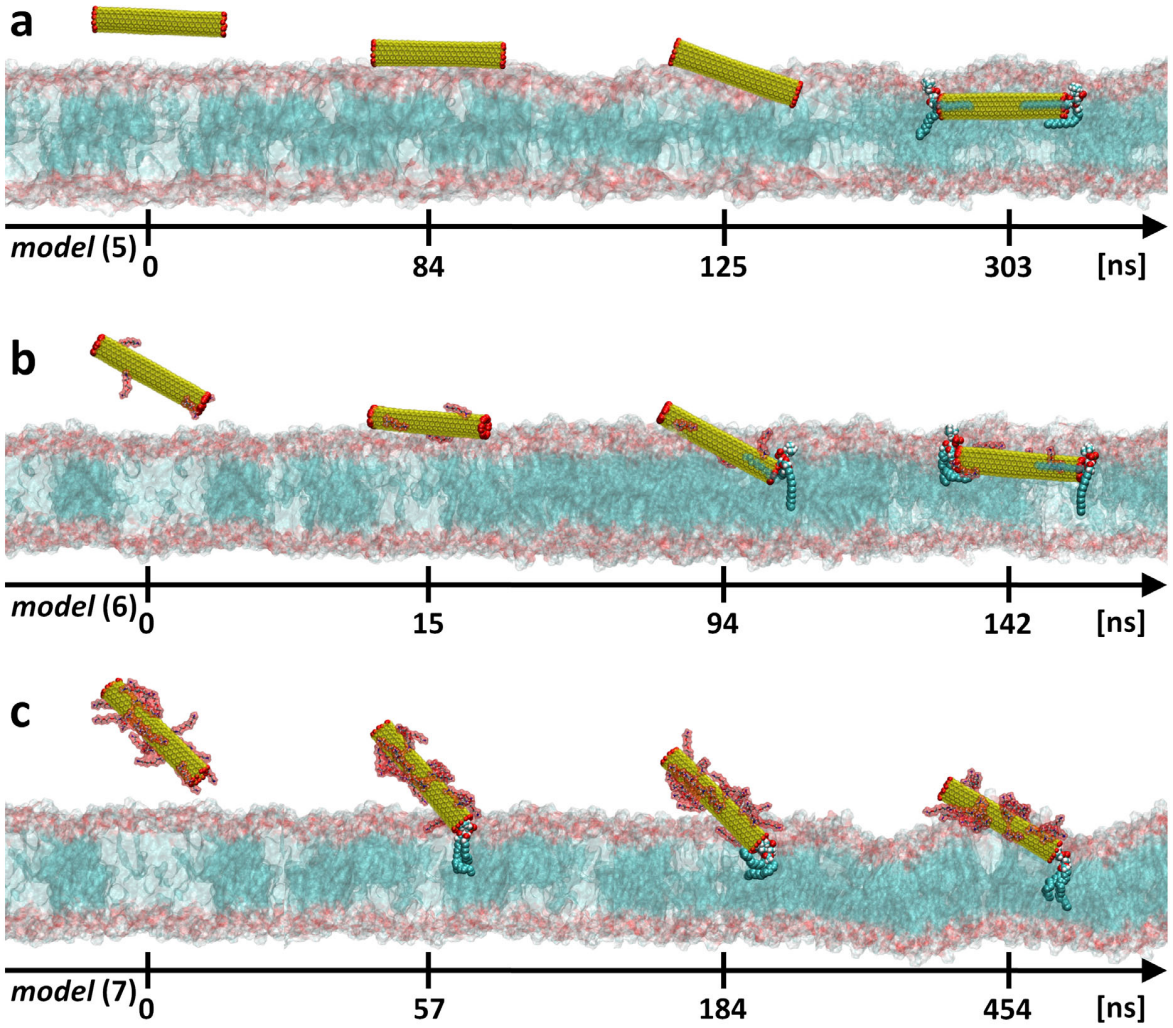
Uptake path of open ended *f*-CNTs. Results obtained from unconstrained MD simulations with: **a,** opened and non functionalized SWNT [model **(5)**]; **b,** low degree functionalized and opened SWNT [model **(6)**]; **c,** or highly functionalized and opened SWNT [model **(7)**]. The yellow surface represents the SWNT core. H atoms (red balls) are used to passivate the SWNT edges. The TEG-NH_3_^+^ functional groups (red surfaces) are attached to the SWNT surface. The lipid membrane head and tails sections are shown as red and blue surfaces, respectively. Note that at the end of each trajectory, a single lipid molecule stays strongly anchored at the SWNT tips. This anchored lipid molecule is shown explicitly as blue (acyl chains), red (carboxyl group) and blue/white (phosphatidylcholine headgroup) balls. For clarity reasons, water molecules and counterions are not shown.

We have here conducted similar MD studies for the opened SWNTs placed near POPC lipid membrane. We did not observe major differences on the overall uptake process during SWNT internalization (Figures 6, S5, S6 and S7): the opened *f*-CNTs diffuse also passively inside the membrane whatever their degree of functionalization. Consistently with the closed configurations, the landing process was observed only for the neat (see Figure S5) or low degree functionalized (Figure S6) opened SWNTs. The only noticeable difference between opened and closed nanotubes concerns the penetration phase. In many aspects this translocation is peculiar. The opened nanotube interior provides for the possibility that water molecules or either lipid headgroups or lipid tails penetrate their exposed extremity, as already observed. [20], [28] Accordingly, the nanotubes during their translocation can drag such stuck lipids resulting in an important rearrangement of the local structure of the bilayer. This behavior was recently reported in two examples. [20], [27] Such a scenario is shown to substantially increase the free energy barrier to translocation with respect to the closed nanotube case. Finally, the strong attractive membrane−/open-SWNT/interactions provide also a decrease of the local area per lipid headgroup by dragging the whole SWNT parallel to the membrane surface during immersion, as recently revealed using coarse grained molecular dynamics approach. [28].

### Conclusions

In summary, unconstrained MD simulations on different models of closed and opened functionalized or non functionalized SWNTs reveal a large propensity for passive transport across phospholipid membranes. This work reinforces the experimental evidence that short TEG-NH_3_^+^ functionalized SWNTs penetrate via a passive diffusion pathway. A noticeable feature of the uptake mechanism is that it takes place following three distinct consecutive steps: i) landing and floating of the SWNTs on the membrane surface; ii) penetration of the lipid headgroups; and iii) sliding through the lipid tails. Hence, the molecular level study carried herein provides strong support of such mechanism evidenced for non-phagocytic cells using 3D electron tomography imaging. [38].

A second main feature is that functionalized SWNTs undergo also a nanoneedle-like diffusion mechanism through membranes. However, the study revealed that the degree of functionalization influences slightly the two first steps and seems to specifically determine the SWNTs membranes penetration angle. Finally, apart from the opened SWNTs which likely drag a lipid molecule during the internalization process, no noticeable modification of the lipid bilayer overall structure was observed.

We may therefore conclude from this extensive computational study that direct insertion seems to be in play for the short length SWNTs internalization by cell membranes, in contrast to what seems to be the case predominantly for CNTs in the µm range length. [12], [39], [40].

Statistical approaches using for instance Monte Carlo simulations could shed light on the permeation process for much longer (sub-µm length) nanotubes. At any rate much further studies are still needed in order to analyze the SWNT length dependence of the uptake mechanism.

## Methods

### Molecular Dynamics Methods and Protocols

Unconstrained full atomistic MD simulations were performed using the equilibrated, fully hydrated palmitoyl-oleoyl-phosphatidylcholine (POPC) membrane model with a total simulation time reaching 3.35 µs. We considered the system consisting of 180 POPC units, 14,650 water molecules and one of the following SWNT models (Figure 1 and Figure 5) characterized by a length of 5 nm and a diameter of ∼1 nm:

1. non functionalized closed (6,6)-SWNT [simulation time of 509 ns]
2. closed (6,6)-SWNT functionalized at its sidewall with 7 ammonium groups [simulation time of 423 ns]
3. closed (6,6)-SWNT functionalized at its sidewall and tips with 7 ammonium groups (2 on each tip and 3 on the sidewall) [simulation time of 395 ns]
4. closed (6,6)-SWNT functionalized at its sidewall with 20 ammonium groups [simulation time of 779 ns]
5. non functionalized opened (6,6)-SWNT explicitly protonated at the edges [simulation time of 443 ns], or **(5′)** non explicitly protonated at the edges [simulation time of 200 ns]; the latter was not represented in any of the figures
6. opened (6,6)-SWNT non explicitly protonated at the edges and functionalized with 4 ammonium groups [simulation time of 142 ns]
7. opened (6,6)-SWNT explicitly protonated at the edges and functionalized with 20 ammonium groups [simulation time of 466 ns]

The ammonium groups at the end of triethylene glycol chain (TEG-NH_3_^+^) were randomly dispersed on the whole surface of the nanotube in order to keep the distance between them as far as possible. The necessary simulation parameters where obtained as described below. The differences between explicit and non explicit H ends of the open nanotubes are presented at the end of this section. The initial dimensions of the simulation cells of 70×74×124 Å^3^ were chosen to provide for a large enough water layer (86 Å) (taking account periodic boundary conditions) to accommodate the 53 Å long SWNTs.

All MD simulations have been carried out in the NPT ensemble (constant Number of particles, Pressure and Temperature) using NAMD2.7b2, [41] a program targeted for massively parallel architectures. Short- and long-range forces were calculated every 1 and 2 time-steps, respectively, with a time step of 2.0 fs. Long-range electrostatic forces were taken into account using the particle mesh Ewald (PME) approach. [42] The Langevin dynamics algorithm and the Langevin piston Nosé-Hoover method [43] were used to maintain 300 K temperature and 1 atm pressure in the system. At the temperature set for the study, the bilayer is in the biologically relevant liquid crystal Lα phase.

The force field parameters for lipid were taken from CHARMM27 [44] with the united atoms extension for acyl chains (hydrogen atoms assumed to carbon atoms). The intra- and intermolecular potentials for water were taken from the TIP3P model. [45].

The force field used here for lipid membranes provide structural results for hydrated POPC that are in good agreement with experiments and with previous simulations. Indeed the area per lipid and headgroup to headgroup distances (P-P) obtained in our simulations (respectively 64.9±1.4 Å^2^ and 42±0.5 Å) compare well with previously reported data (*cf.* [46] for a recent review).

For the carbon-carbon or carbon-water interactions in SWNTs, we follow the Bedrov description of Lennard-Jones potential [47], [48], [49] with σ_CC_ = 3.895 Å, ε_CC_ = 0.276 kJ mol^−1^ and σ_CO_ = 3.580 Å, ε_CO_ = 0.392 kJ mol^−1^. For the necessary potential parameters of the amino derivatives we followed the general procedure as described by Norrby and Brandt [50] based on construction of the Hessian matrix (the matrix of second derivatives of the energy with respect to geometry) for further use in the force field parameterization.

### Quantum Calculations

The geometrical optimization of a single amino derivative was performed using the Hartree-Fock approach with polarized continuum water model using integral equation formalism (IEFPCM) able to reproduce environmental effect of the solvent. The split-valence 6-31+G basis set was employed for all atoms and obtained Mulliken partial charges were applied to the molecular model. [51] We decided to employ the Mulliken charges over the other types in order to be consistent with partial atomic charges of the CHARMM27 force field, which are initially obtained from a Mulliken population analysis.

Additionally, open nanotube’s edges are fitted with the specific Mulliken partial charges distributions, according to the results obtained from the quantum mechanics calculations with the same level of theory as for NH_3_^+^ parts (HF/6-31+G and with IEFPCM). This procedure gives a supplementary (and naturally present due to carbon polarizability) charge-based functionalization inducing local electrostatic force coming from edge dipole moments. For models **(5)’** and **(6)** when the hydrogen atoms were assumed to carbon atoms (as in the case of lipid acyl chains) the edge dipole moment is equal to 5 D. For explicitly protonated open SWNT **(5)** and **(7)**, the edge dipole moment rise to 13.5 D. SWNT carbon atoms other than in open edges do not carry charges.

Evaluation of donor/acceptor distance between nitrogen and phosphorous coming from functionnal group and lipid headgroup, respectively, where evaluated employing two different levels of theory *ie.* HF/6-31+G(d,p) and B3LYP/6-31+G(d,p), each with IEFPCM approach. TEG-NH_3_^+^ functionalization was mimicked by ethanolammonium ion and phosphocholine was used as a simplified model of the POPC lipid molecule (see also [36]). All the *ab-initio* quantum calculations were performed using Gaussian 03 package software. [52].

### Voronoi Tessellation Method

We use 2D tessellations with Voronoi polyhedra [53] to study local instantaneous changes in the area per headgroup of the lipids in the vicinity of the SWNT tube, for the centers of mass of each lipid phosphorous, using custom scripting.

### Adaptive Biasing Force (ABF) Method

The free energy profile was estimated using GPU accelerated NAMD 2.9b3 software [41] with the ABF extensions integrated in the Collective Variables module [54] and under the same conditions as described for the MD simulations. In order to sample the SWNT insertion over the trajectory observed during unconstrained MD simulations we extracted the configurations from previous MD run of model **(1),** then the sampling using 2 Å windows superposed at each 1 Å was performed. The minimal sampling was equal to 100’000 samples for each step along reaction coordinate under study, taken as a distance between center of mass (COM) of the non functionalized closed SWNT **(1)** and the COM of the lipid bilayer along the z-axis, with the step of 0.1 Å.

## Supporting Information

Figure S1 Low degree surface functionalized and closed SWNT [model **(2)**] presents 3-step insertion. **a,** Landing, penetration and sliding phases into POPC lipid bilayer and **b,** corresponding Voronoi tessellations of membrane surface are presented. **c,** 3-step insertion trajectory as a function of unconstrained simulation time, with membrane thickness control (left ordinate scale) and attack angle with respect to the normal of the membrane plane (right ordinate scale). Color codes: **a,** SWNT position is indicated by yellow surface, with red (charged) amino groups. Lipid’s nitrogen, phosphate groups and hydrocarbon tails are blue, red and cyan surfaces, respectively. For clarity reasons, water molecules from the system are not shown. **b,** Red areas in Voronoi diagrams correspond to internalizing SWNT. **c,** Left ordinate scale refer to SWNT center of mass position (black curve), mean nitrogen position of lipid headgroups (red curve), mean phosphorous position of lipid headgroups (blue curve) and mean position of lipid glycerol backbone (green curve). Right ordinate scale refers to SWNT insertion angle (α) with respect to the normal of the membrane plane (wine curve). The angle curve is smoothed by averaging the angle value in 1 ns window.
(TIF)

Figure S2 Low degree surface and edges functionalized and closed SWNT [model **(3)**] presents 3-step insertion. **a,** Landing, penetration and sliding phases into POPC lipid bilayer and **b,** corresponding Voronoi tessellations of membrane surface are presented. **c,** 3-step insertion trajectory as a function of unconstrained simulation time, with membrane thickness control (left ordinate scale) and attack angle with respect to the normal of the membrane plane (right ordinate scale). Color codes: **a,** SWNT position is indicated by yellow surface, with red (charged) or green (deprotonated) amino groups. Lipid’s nitrogen, phosphate groups and hydrocarbon tails are blue, red and cyan surfaces, respectively. For clarity reasons, water molecules from the system are not shown. **b,** Red areas in Voronoi diagrams correspond to internalizing SWNT. **c,** Left ordinate scale refer to SWNT center of mass position (black curve), mean nitrogen position of lipid headgroups (red curve), mean phosphorous position of lipid headgroups (blue curve) and mean position of lipid glycerol backbone (green curve). Right ordinate scale refers to SWNT insertion angle (α) with respect to the normal of the membrane plane (wine curve). The angle curve is smoothed by averaging the angle value in 1 ns window.
(TIF)

Figure S3 High degree surface functionalized SWNT [model **(4)**] presents 3-step insertion. **a,** vertical landing (instead of landing and floating), perpendicular penetration and sliding phases into POPC lipid bilayer. **b,** corresponding Voronoi tessellations of membrane surface are presented. **c,** 3-step insertion trajectory as a function of unconstrained simulation time, with membrane thickness control (left ordinate scale) and attack angle with respect to the normal of the membrane plane (right ordinate scale). Color codes: **a,** SWNT position is indicated by yellow surface, with red (charged) or green (deprotonated) amino groups. Lipid’s nitrogen, phosphate groups and hydrocarbon tails are blue, red and cyan surfaces, respectively. For clarity reasons, water molecules from the system are not shown. **b,** Red areas in Voronoi diagrams correspond to internalizing SWNT. **c,** Left ordinate scale refer to SWNT center of mass position (black curve), mean nitrogen position of lipid headgroups (red curve), mean phosphorous position of lipid headgroups (blue curve) and mean position of lipid glycerol backbone (green curve). Right ordinate scale refers to SWNT insertion angle (α) with respect to the normal of the membrane plane (wine curve). The angle curve is smoothed by averaging the angle value in 1 ns window.
(TIF)

Figure S4 Example of distance distribution between nitrogen from TEG-NH_3_^+^ functional group and neighbor phosphorous belonging to lipid headgroup. Data corresponds to one of the functional groups from SWNT [model **(7)**] and for all 466 ns of simulation.
(TIF)

Figure S5 Opened SWNT [model **(5)**] presents 3-step insertion. **a,** Landing, penetration and sliding phases into POPC lipid bilayer and **b,** corresponding Voronoi tessellations of membrane surface are presented. **c,** 3-step insertion trajectory as a function of unconstrained simulation time, with membrane thickness control (left ordinate scale) and attack angle with respect to the normal of the membrane plane (right ordinate scale). Color codes: **a,** SWNT position is indicated by yellow surface, and passivated edges are shown as red balls. Lipid’s nitrogen, phosphate groups and hydrocarbon tails are blue, red and cyan surfaces, respectively. For clarity reasons, water molecules from the system are not shown. **b,** Red areas in Voronoi diagrams correspond to internalizing SWNT. **c,** Left ordinate scale refer to SWNT center of mass position (black curve), mean nitrogen position of lipid headgroups (red curve), mean phosphorous position of lipid headgroups (blue curve) and mean position of lipid glycerol backbone (green curve). Right ordinate scale refers to SWNT insertion angle (α) with respect to the normal of the membrane plane (wine curve). The angle curve is smoothed by averaging the angle value in 1 ns window.
(TIF)

Figure S6 Low degree surface functionalized and opened SWNT [model **(6)**] presents 3-step insertion. **a,** Landing, penetration and sliding phases into POPC lipid bilayer and **b,** corresponding Voronoi tessellations of membrane surface are presented. **c,** 3-step insertion trajectory as a function of unconstrained simulation time, with membrane thickness control (left ordinate scale) and attack angle with respect to the normal of the membrane plane (right ordinate scale). Color codes: **a,** SWNT position is indicated by yellow surface, with red (charged) amino groups. SWNT passivated edges are shown as red balls. Lipid’s nitrogen, phosphate groups and hydrocarbon tails are blue, red and cyan surfaces, respectively. For clarity reasons, water molecules from the system are not shown. **b,** Red areas in Voronoi diagrams correspond to internalizing SWNT. **c,** Left ordinate scale refer to SWNT center of mass position (black curve), mean nitrogen position of lipid headgroups (red curve), mean phosphorous position of lipid headgroups (blue curve) and mean position of lipid glycerol backbone (green curve). Right ordinate scale refers to SWNT insertion angle (α) with respect to the normal of the membrane plane (wine curve). The angle curve is smoothed by averaging the angle value in 1 ns window.
(TIF)

Figure S7 High degree surface functionalized and opened SWNT [model **(7)**] presents incomplete insertion. **a,** Only landing and penetration phases into POPC lipid bilayer occurs. **b,** Corresponding Voronoi tessellations of membrane surface are presented. **c,** 2-step insertion trajectory as a function of unconstrained simulation time, with membrane thickness control (left ordinate scale) and attack angle with respect to the normal of the membrane plane (right ordinate scale). Color codes: **a,** SWNT position is indicated by yellow surface, with red (charged) amino groups. SWNT passivated edges are shown as red balls. Lipid’s nitrogen, phosphate groups and hydrocarbon tails are blue, red and cyan surfaces, respectively. For clarity reasons, water molecules from the system are not shown. **b,** Red areas in Voronoi diagrams correspond to internalizing SWNT. **c,** Left ordinate scale refer to SWNT center of mass position (black curve), mean nitrogen position of lipid headgroups (red curve), mean phosphorous position of lipid headgroups (blue curve) and mean position of lipid glycerol backbone (green curve). Right ordinate scale refers to SWNT insertion angle (α) with respect to the normal of the membrane plane (wine curve). The angle curve is smoothed by averaging the angle value in 1 ns window.
(TIF)

Video S1 Video shows the passive incorporation of neat closed SWNT [model **(1)**] into POPC lipid bilayer and corresponds to the first 170 ns of the trajectory presented in Fig. 2a. SWNT position is indicated by yellow surface. Lipid’s nitrogen, phosphate groups and hydrocarbon tails are blue, red and cyan surfaces, respectively. For clarity reasons, water molecules from the system are not shown.
(FLV)

Video S2 Video shows the incorporation of *f*-CNT with 7 amino derivatives present on sidewall and on tips [model **(3)**] into POPC lipid bilayer and corresponds to all the 395 ns of calculated trajectory presented in Fig. 4b. SWNT position is indicated by yellow surface, with red (charged) or green (deprotonated) amino groups. Lipid’s nitrogen, phosphate groups and hydrocarbon tails are blue, red and cyan surfaces, respectively. For clarity reasons, water molecules from the system are not shown.
(FLV)

Video S3 Video shows the incorporation of *f*-CNT with 20 amino derivatives [model **(4)**] into POPC lipid bilayer and corresponds to all the 779 ns of calculated trajectory presented in Fig. 4c. SWNT position is indicated by yellow surface, with red (charged) or green (deprotonated) amino groups. Lipid’s nitrogen, phosphate groups and hydrocarbon tails are blue, red and cyan surfaces, respectively. For clarity reasons, water molecules from the system are not shown.
(FLV)
