# Supplementary figures and images for: Insertion of Short Amino-Functionalized Single-Walled Carbon Nanotubes into Phospholipid Bilayer Occurs by Passive Diffusion
Source: PLoS One. 2012 Jul 16;7(7):e40703. doi: 10.1371/journal.pone.0040703 (PMC3398044; doi:10.1371/journal.pone.0040703)

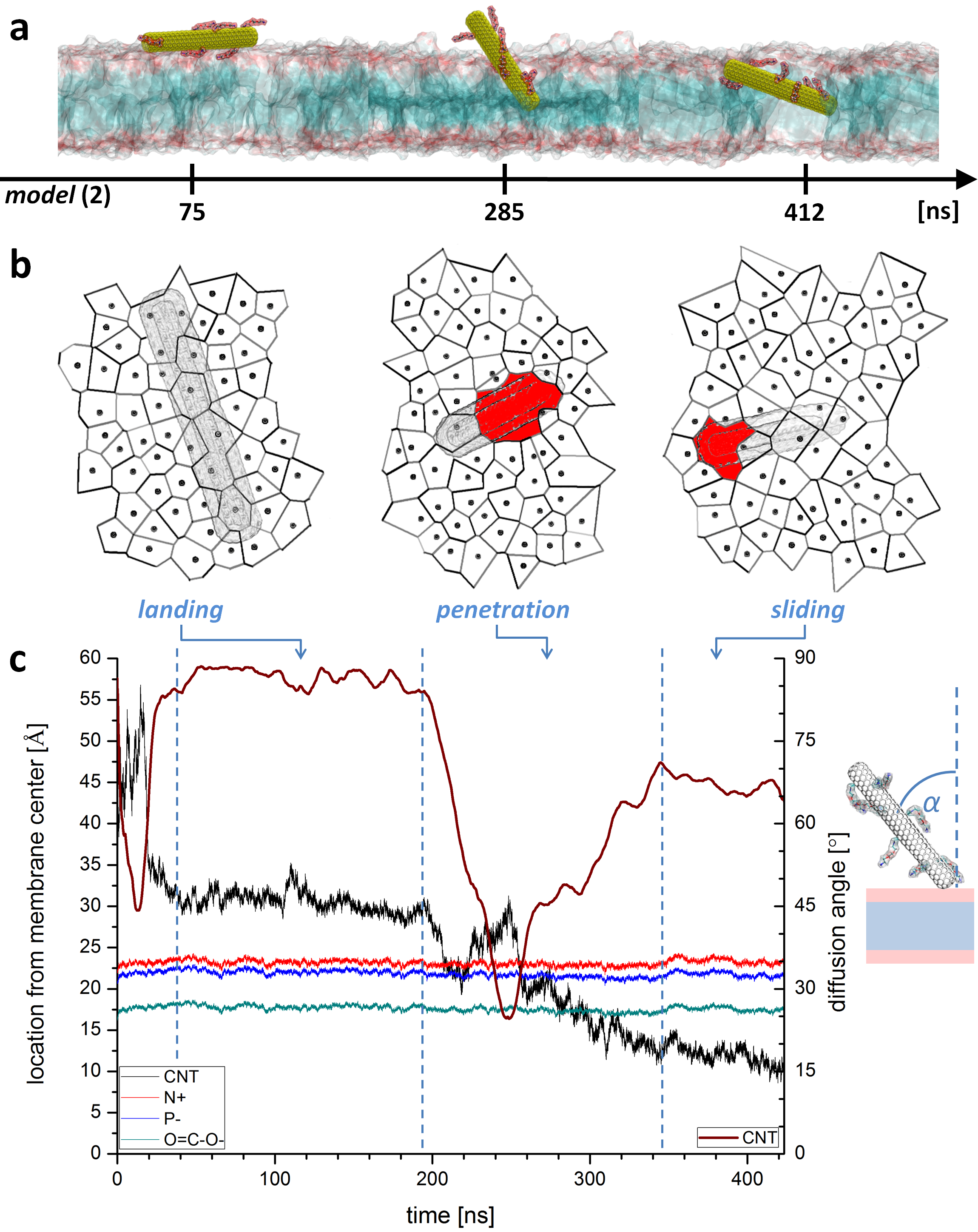

Supplement: Figure S1 — Low degree surface functionalized and closed SWNT [model (2)] presents 3-step insertion. a, Landing, penetration and sliding phases into POPC lipid bilayer and b, corresponding Voronoi tessellations of membrane surface are presented. c, 3-step insertion trajectory as a function of unconstrained simulation time, with membrane thickness control (left ordinate scale) and attack angle with respect to the normal of the membrane plane (right ordinate scale). Color codes: a, SWNT position is indicated by yellow surface, with red (charged) amino groups. Lipid’s nitrogen, phosphate groups and hydrocarbon tails are blue, red and cyan surfaces, respectively. For clarity reasons, water molecules from the system are not shown. b, Red areas in Voronoi diagrams correspond to internalizing SWNT. c, Left ordinate scale refer to SWNT center of mass position (black curve), mean nitrogen position of lipid headgroups (red curve), mean phosphorous position of lipid headgroups (blue curve) and mean position of lipid glycerol backbone (green curve). Right ordinate scale refers to SWNT insertion angle (α) with respect to the normal of the membrane plane (wine curve). The angle curve is smoothed by averaging the angle value in 1 ns window. (TIF) [file pone.0040703.s001.tif]

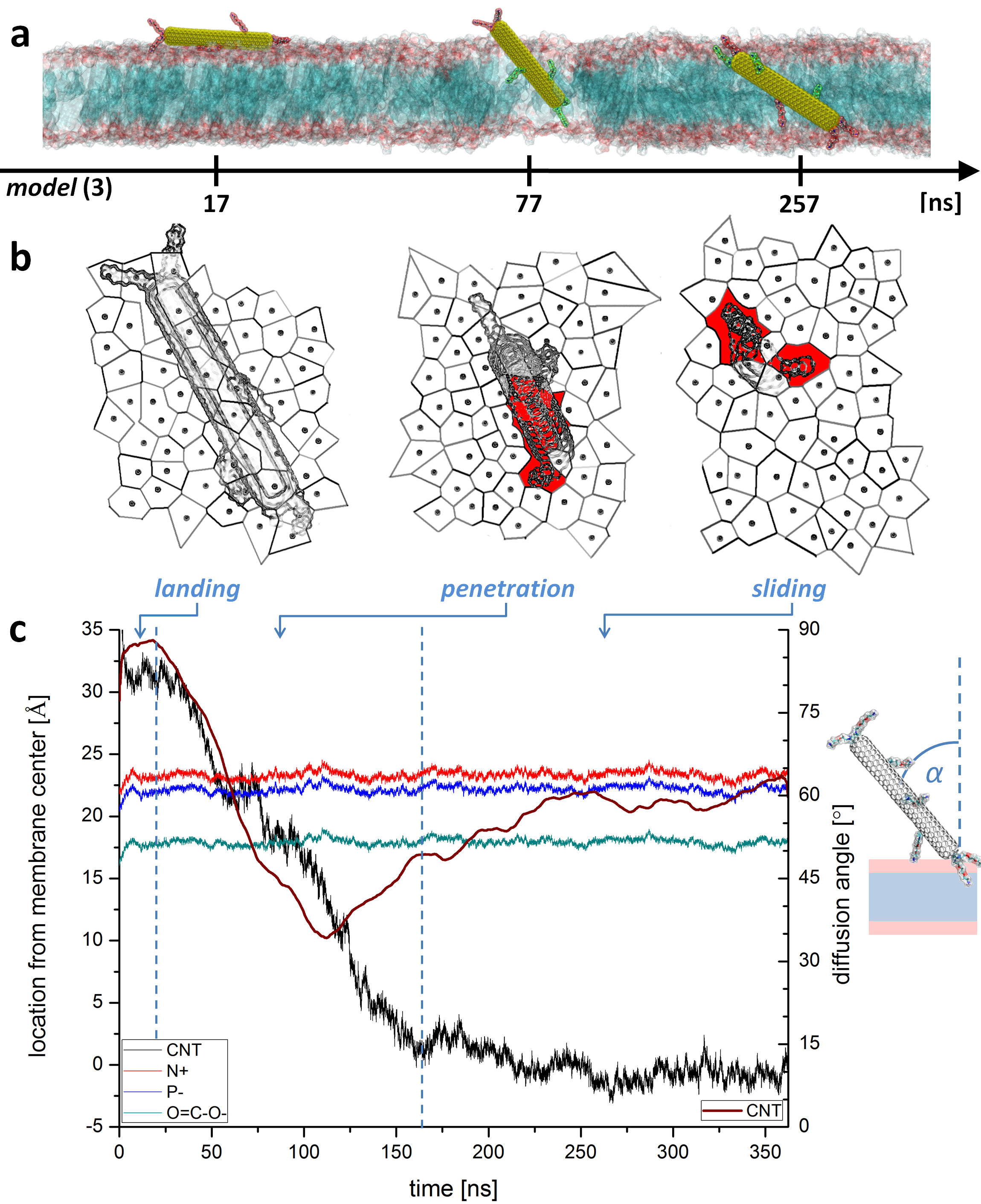

Supplement: Figure S2 — Low degree surface and edges functionalized and closed SWNT [model (3)] presents 3-step insertion. a, Landing, penetration and sliding phases into POPC lipid bilayer and b, corresponding Voronoi tessellations of membrane surface are presented. c, 3-step insertion trajectory as a function of unconstrained simulation time, with membrane thickness control (left ordinate scale) and attack angle with respect to the normal of the membrane plane (right ordinate scale). Color codes: a, SWNT position is indicated by yellow surface, with red (charged) or green (deprotonated) amino groups. Lipid’s nitrogen, phosphate groups and hydrocarbon tails are blue, red and cyan surfaces, respectively. For clarity reasons, water molecules from the system are not shown. b, Red areas in Voronoi diagrams correspond to internalizing SWNT. c, Left ordinate scale refer to SWNT center of mass position (black curve), mean nitrogen position of lipid headgroups (red curve), mean phosphorous position of lipid headgroups (blue curve) and mean position of lipid glycerol backbone (green curve). Right ordinate scale refers to SWNT insertion angle (α) with respect to the normal of the membrane plane (wine curve). The angle curve is smoothed by averaging the angle value in 1 ns window. (TIF) [file pone.0040703.s002.tif]

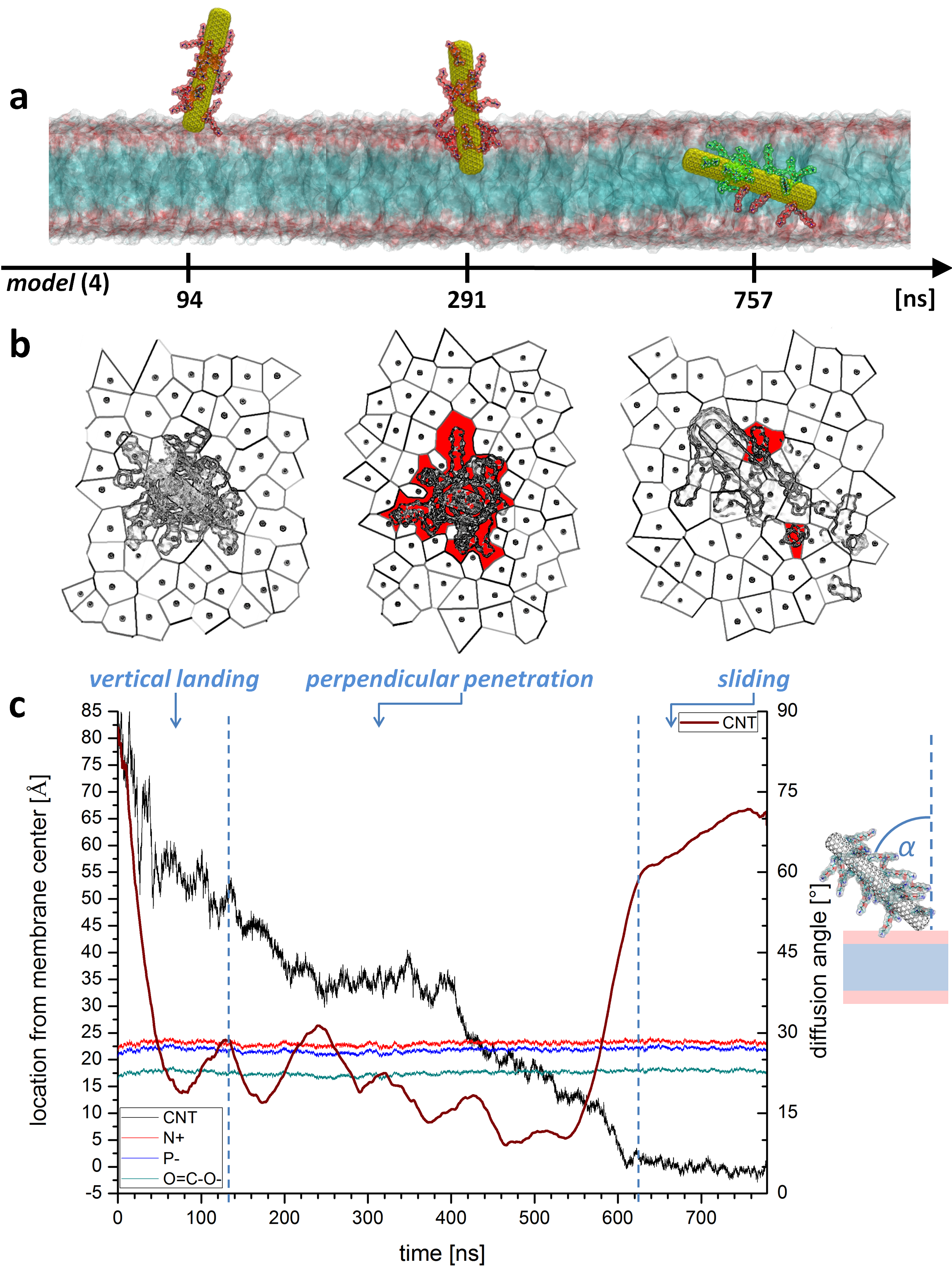

Supplement: Figure S3 — High degree surface functionalized SWNT [model (4)] presents 3-step insertion. a, vertical landing (instead of landing and floating), perpendicular penetration and sliding phases into POPC lipid bilayer. b, corresponding Voronoi tessellations of membrane surface are presented. c, 3-step insertion trajectory as a function of unconstrained simulation time, with membrane thickness control (left ordinate scale) and attack angle with respect to the normal of the membrane plane (right ordinate scale). Color codes: a, SWNT position is indicated by yellow surface, with red (charged) or green (deprotonated) amino groups. Lipid’s nitrogen, phosphate groups and hydrocarbon tails are blue, red and cyan surfaces, respectively. For clarity reasons, water molecules from the system are not shown. b, Red areas in Voronoi diagrams correspond to internalizing SWNT. c, Left ordinate scale refer to SWNT center of mass position (black curve), mean nitrogen position of lipid headgroups (red curve), mean phosphorous position of lipid headgroups (blue curve) and mean position of lipid glycerol backbone (green curve). Right ordinate scale refers to SWNT insertion angle (α) with respect to the normal of the membrane plane (wine curve). The angle curve is smoothed by averaging the angle value in 1 ns window. (TIF) [file pone.0040703.s003.tif]

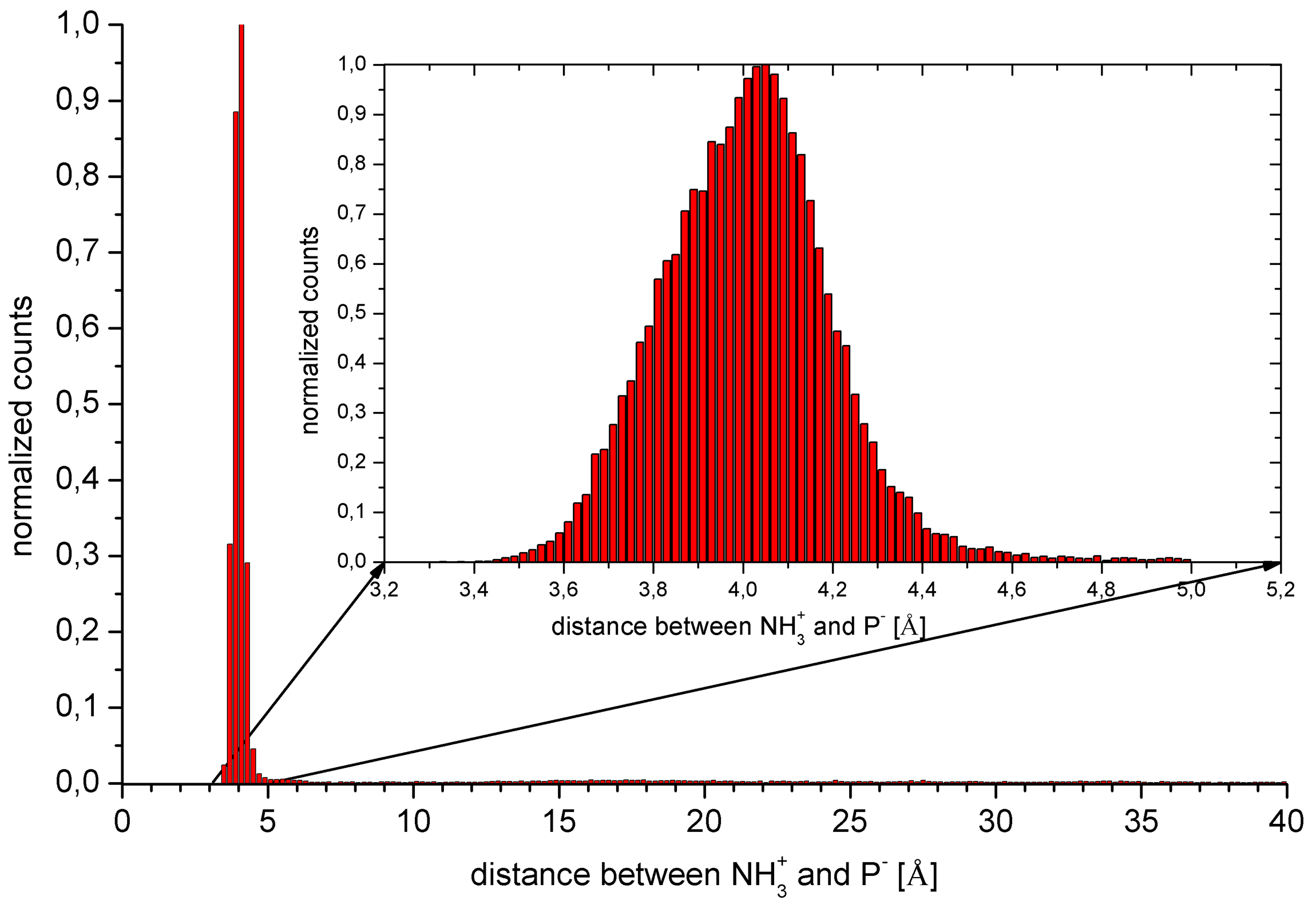

Supplement: Figure S4 — Example of distance distribution between nitrogen from TEG-NH3 + functional group and neighbor phosphorous belonging to lipid headgroup. Data corresponds to one of the functional groups from SWNT [model (7)] and for all 466 ns of simulation. (TIF) [file pone.0040703.s004.tif]

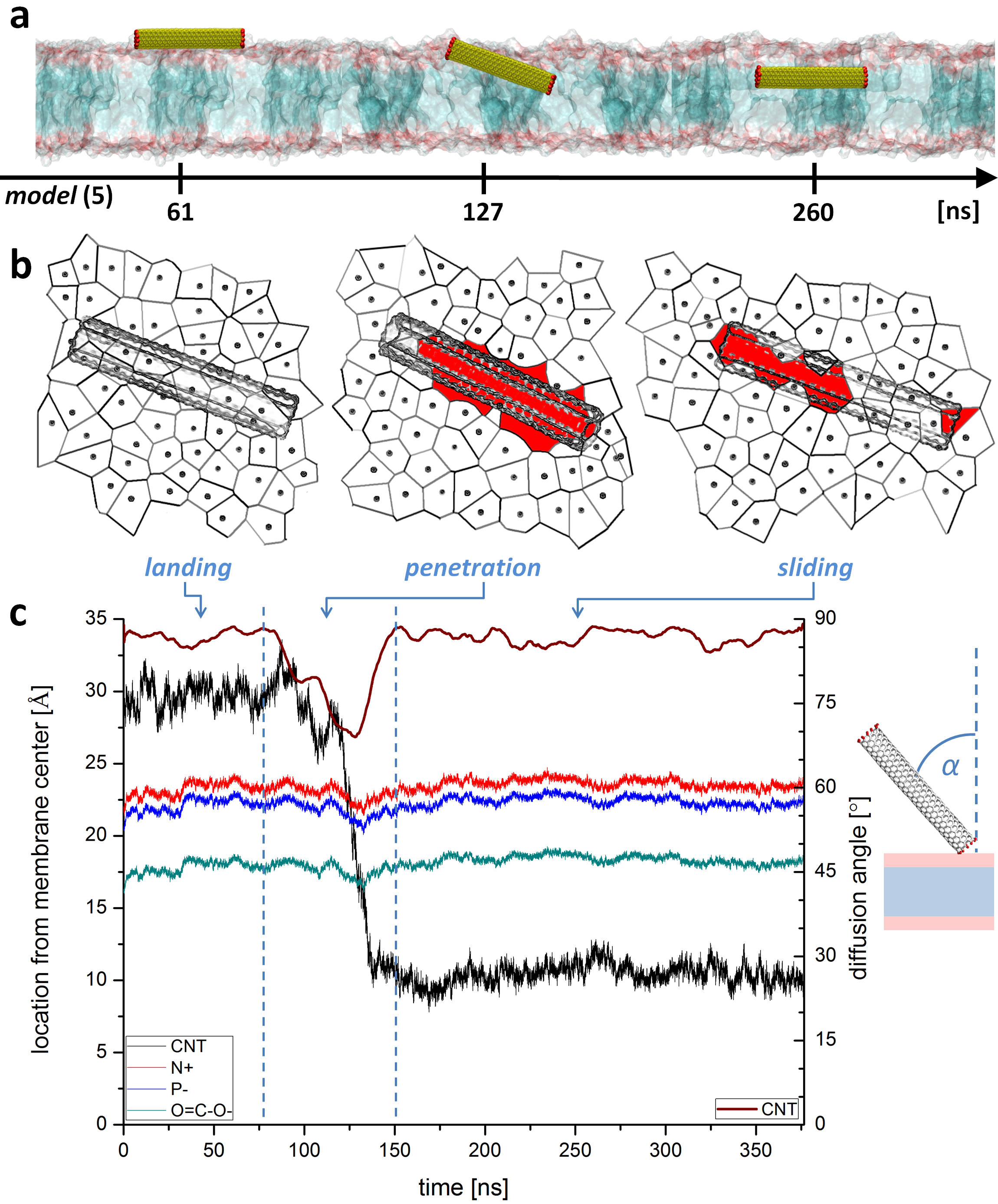

Supplement: Figure S5 — Opened SWNT [model (5)] presents 3-step insertion. a, Landing, penetration and sliding phases into POPC lipid bilayer and b, corresponding Voronoi tessellations of membrane surface are presented. c, 3-step insertion trajectory as a function of unconstrained simulation time, with membrane thickness control (left ordinate scale) and attack angle with respect to the normal of the membrane plane (right ordinate scale). Color codes: a, SWNT position is indicated by yellow surface, and passivated edges are shown as red balls. Lipid’s nitrogen, phosphate groups and hydrocarbon tails are blue, red and cyan surfaces, respectively. For clarity reasons, water molecules from the system are not shown. b, Red areas in Voronoi diagrams correspond to internalizing SWNT. c, Left ordinate scale refer to SWNT center of mass position (black curve), mean nitrogen position of lipid headgroups (red curve), mean phosphorous position of lipid headgroups (blue curve) and mean position of lipid glycerol backbone (green curve). Right ordinate scale refers to SWNT insertion angle (α) with respect to the normal of the membrane plane (wine curve). The angle curve is smoothed by averaging the angle value in 1 ns window. (TIF) [file pone.0040703.s005.tif]

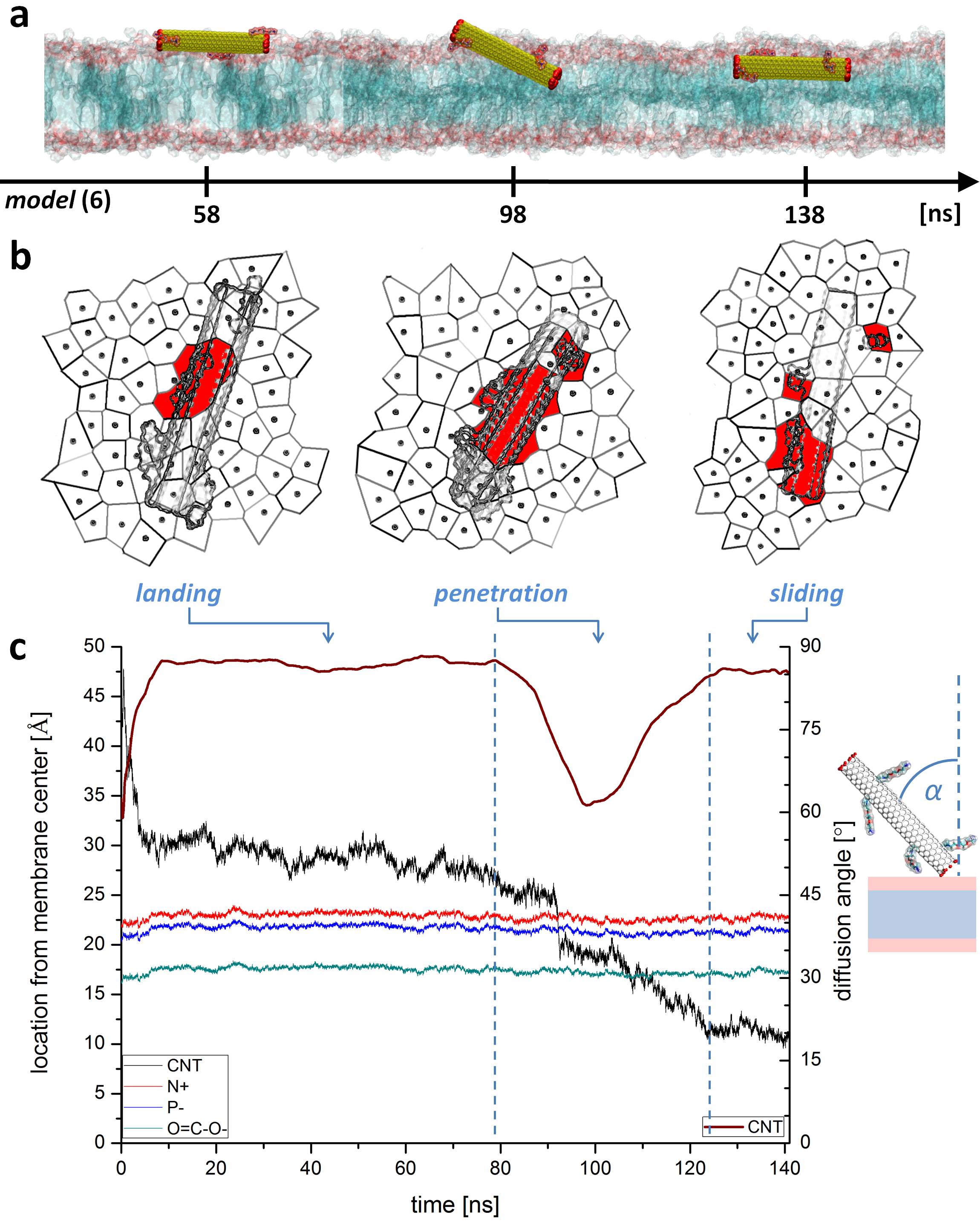

Supplement: Figure S6 — Low degree surface functionalized and opened SWNT [model (6)] presents 3-step insertion. a, Landing, penetration and sliding phases into POPC lipid bilayer and b, corresponding Voronoi tessellations of membrane surface are presented. c, 3-step insertion trajectory as a function of unconstrained simulation time, with membrane thickness control (left ordinate scale) and attack angle with respect to the normal of the membrane plane (right ordinate scale). Color codes: a, SWNT position is indicated by yellow surface, with red (charged) amino groups. SWNT passivated edges are shown as red balls. Lipid’s nitrogen, phosphate groups and hydrocarbon tails are blue, red and cyan surfaces, respectively. For clarity reasons, water molecules from the system are not shown. b, Red areas in Voronoi diagrams correspond to internalizing SWNT. c, Left ordinate scale refer to SWNT center of mass position (black curve), mean nitrogen position of lipid headgroups (red curve), mean phosphorous position of lipid headgroups (blue curve) and mean position of lipid glycerol backbone (green curve). Right ordinate scale refers to SWNT insertion angle (α) with respect to the normal of the membrane plane (wine curve). The angle curve is smoothed by averaging the angle value in 1 ns window. (TIF) [file pone.0040703.s006.tif]

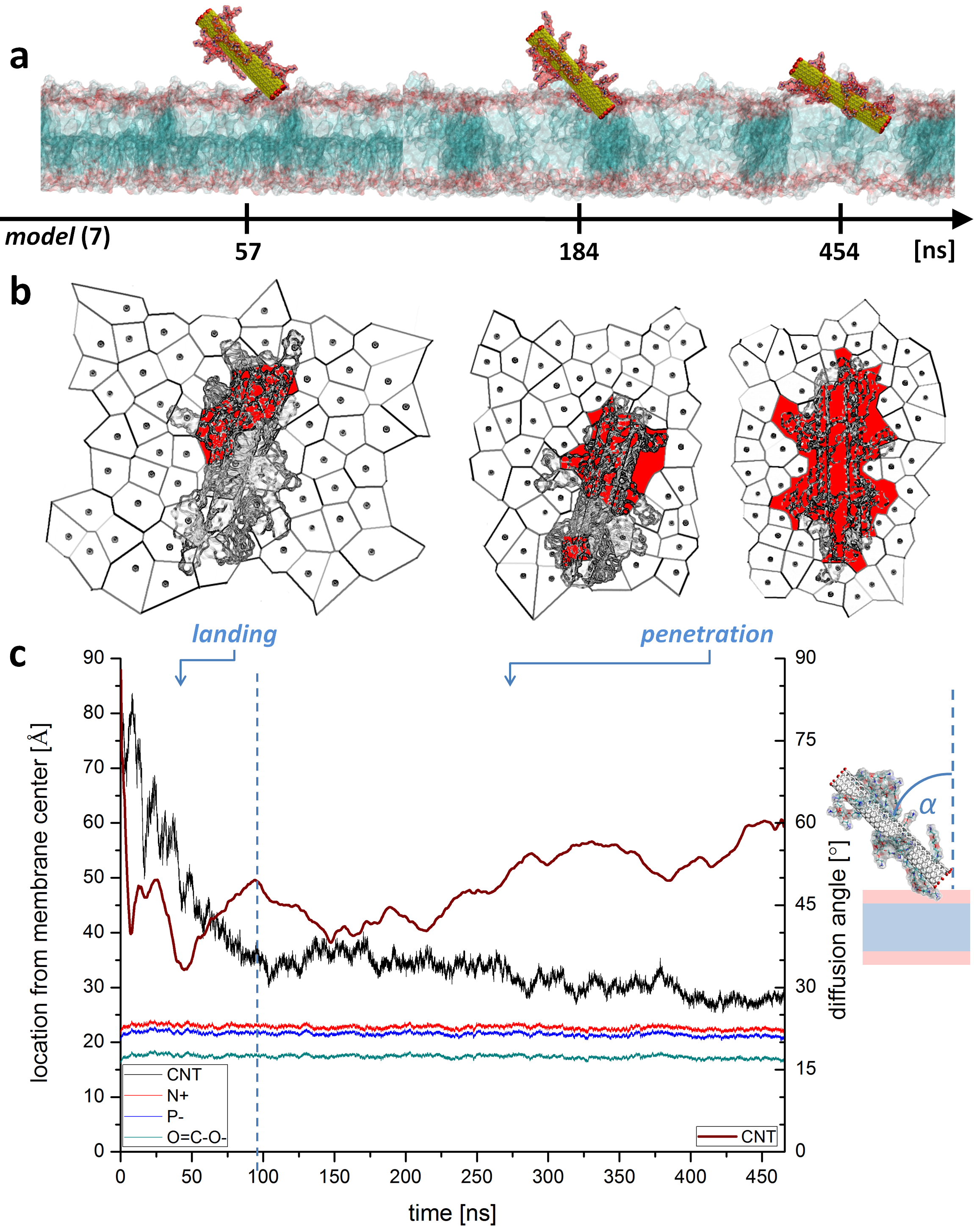

Supplement: Figure S7 — High degree surface functionalized and opened SWNT [model (7)] presents incomplete insertion. a, Only landing and penetration phases into POPC lipid bilayer occurs. b, Corresponding Voronoi tessellations of membrane surface are presented. c, 2-step insertion trajectory as a function of unconstrained simulation time, with membrane thickness control (left ordinate scale) and attack angle with respect to the normal of the membrane plane (right ordinate scale). Color codes: a, SWNT position is indicated by yellow surface, with red (charged) amino groups. SWNT passivated edges are shown as red balls. Lipid’s nitrogen, phosphate groups and hydrocarbon tails are blue, red and cyan surfaces, respectively. For clarity reasons, water molecules from the system are not shown. b, Red areas in Voronoi diagrams correspond to internalizing SWNT. c, Left ordinate scale refer to SWNT center of mass position (black curve), mean nitrogen position of lipid headgroups (red curve), mean phosphorous position of lipid headgroups (blue curve) and mean position of lipid glycerol backbone (green curve). Right ordinate scale refers to SWNT insertion angle (α) with respect to the normal of the membrane plane (wine curve). The angle curve is smoothed by averaging the angle value in 1 ns window. (TIF) [file pone.0040703.s007.tif]
